# The Love and Hate Relationship between T5SS and Other Secretion Systems in Bacteria

**DOI:** 10.3390/ijms25010281

**Published:** 2023-12-24

**Authors:** Yi Luo, Ziyue Chen, Siqi Lian, Xingduo Ji, Chunhong Zhu, Guoqiang Zhu, Pengpeng Xia

**Affiliations:** 1 College of Veterinary Medicine (Institute of Comparative Medicine), Yangzhou University, Yangzhou 225009, China; luoyi_0910@163.com (Y.L.); chenziyue1122@yeah.net (Z.C.); mx120180715@yzu.edu.cn (S.L.); l15852878389@163.com (X.J.); yzgqzhu@yzu.edu.cn (G.Z.); 2 Jiangsu Co-Innovation Center for Prevention and Control of Important Animal Infectious Diseases and Zoonoses, Yangzhou 225009, China; 3 International Research Laboratory of Prevention and Control of Important Animal Infectious Diseases and Zoonotic Diseases of Jiangsu Higher Education Institutions, Yangzhou University, Yangzhou 225009, China; 4 Jiangsu Institute of Poultry Science, Yangzhou 225009, China; zhuch_1304428@126.com

**Keywords:** secretion systems, T5SS, competition, cooperation, inhibition, autotransporter

## Abstract

Bacteria have existed on Earth for billions of years, exhibiting ubiquity and involvement in various biological activities. To ensure survival, bacteria usually release and secrete effector proteins to acquire nutrients and compete with other microorganisms for living space during long-term evolution. Consequently, bacteria have developed a range of secretion systems, which are complex macromolecular transport machines responsible for transporting proteins across the bacterial cell membranes. Among them, one particular secretion system that stands out from the rest is the type V secretion system (T5SS), known as the “autotransporter”. Bacterial activities mediated by T5SS include adherence to host cells or the extracellular matrix, invasion of host cells, immune evasion and serum resistance, contact-dependent growth inhibition, cytotoxicity, intracellular flow, protease activity, autoaggregation, and biofilm formation. In a bacterial body, it is not enough to rely on T5SS alone; in most cases, T5SS cooperates with other secretion systems to carry out bacterial life activities, but regardless of how good the relationship is, there is friction between the secretion systems. T5SS and T1SS/T2SS/T3SS/T6SS all play a synergistic role in the pathogenic processes of bacteria, such as nutrient acquisition, pathogenicity enhancement, and immune modulation, but T5SS indirectly inhibits the function of T4SS. This could be considered a love–hate relationship between secretion systems. This paper uses the systematic literature review methodology to review 117 journal articles published within the period from 1995 to 2024, which are all available from the PubMed, Web of Science, and Scopus databases and aim to elucidate the link between T5SS and other secretion systems, providing clues for future prevention and control of bacterial diseases.

## 1. Introduction

In nature, many microorganisms cause diseases in humans and animals. Among these microorganisms, bacteria pose a great threat to human and animal health. Bacteria exhibit a complex series of biological activities, including invasion of cells, colonization, and multiplication, to cause diseases in the host [1]. To compete with other microorganisms, acquire nutrition, and evade the host defense system, bacteria secrete effectors [2], but sometimes, these effectors are defended against by the host immune system, so bacteria have evolved a series of secretion systems, including the 11 secretion systems and the chaperone–usher (C-U) pathway [3]. The bacterial secretion systems are sophisticated molecular machines that allow bacteria to transport a variety of different effectors (macromolecules, toxins) without depending on any changes in membrane topology [4].

To date, nine types of secretion systems (T1SS–T9SS) have been extensively studied. Studies on the type X SS (T10SS) and T11SS are scarce, so they are not described in this paper. The secreted effectors must pass through one or two bacterial membranes [5]. Gram-positive bacteria have only an inner membrane (IM), so the transport route is relatively simple. Gram-negative bacteria have both an IM and an outer membrane (OM), which are separated by a peptidoglycan layer [6]. The OM can protect bacteria by colonizing the host cell, evading the host immune system’s defenses, and resisting antibiotics. However, the OM is also a barrier for bacteria to secrete proteins. Gram-negative bacteria have evolved a series of effective mechanisms to transport substances from the cytoplasm to the extracellular space [7]. In Gram-negative bacteria, secretion systems can be classified into two categories. Effectors can cross the two membranes in one step with the help of T1SS, T3SS, T4SS, T6SS, through which they are transported directly from the bacterial cytoplasm to the extracellular space or target cells. Otherwise, the effectors secreted through the common general secretory/twin-arginine translocation (Sec/TAT) pathway first stay in the periplasmic space between the IM and OM for some time, and then, T2SS, T5SS, T8SS, and T9SS, as well as the C-U pathway, help in their secretion into the extracellular space [8,9]. T5SS is a special autotransporter secretion system [10]. In this review, we provide a concise overview of the structures and mechanisms underlying the nine types of secretion systems, with a specific focus on the cooperation, competition, and other interactions between T5SS and other secretion systems in bacterial biological activities.

## 2. Secretion Systems

Upon infection or invasion of the host, secretory proteins are delivered to the surface of the cell or injected directly into the target cell. Bacteria use two pathways to transfer proteins between the IM and OM: the Sec pathway and the TAT system. Bacteria have evolved numerous translocation systems that accommodate a highly diverse array of substrates of varying size, hydrophilicity, structure, and function [11]. In addition, the type IV pilus assembly pathway (T4P) and T2SS have high structural and functional similarities, and the phylogeny of T4P is highly related to that of T2SS. However, the difference between T4P and T2SS is that T4P has a pilus encoded by several major genes, such as *PilA1* and *PilT*. T4P reduces the distance between bacteria and host cells through a pilus stretching and retraction mechanism and promotes bacterial adhesion and biofilm formation [12].

**Figure 1:**
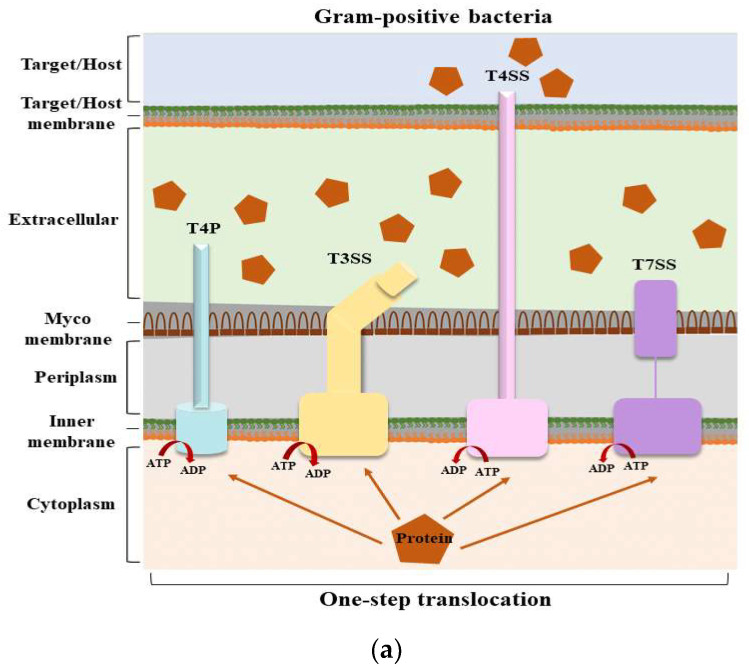

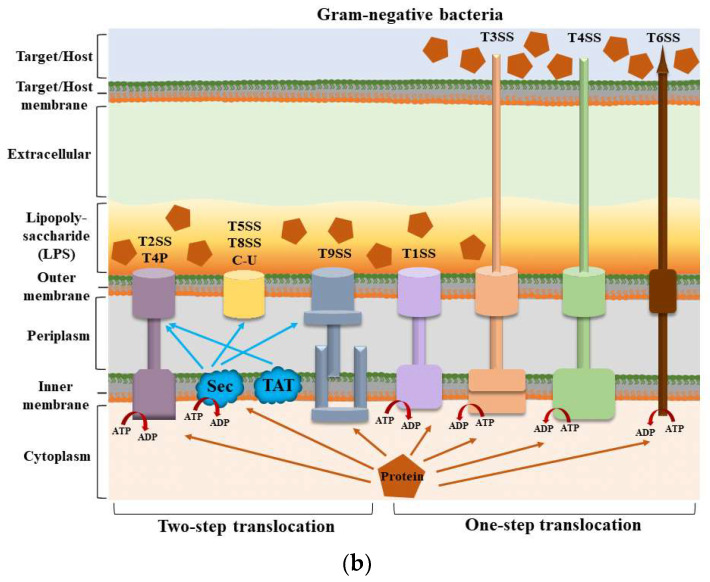
Structures of secretion systems and secretion steps in bacteria. Nine secretion systems (T1SS–T9SS) have been identified in bacteria, including the C-U system and T4P assembly pathway. Some secretion systems are present in both Gram-positive and Gram-negative bacteria. (**a**) The secretion systems of Gram-positive bacteria and the secretion steps; (**b**) The secretion systems of Gram-negative bacteria and the secretion steps. According to their secretion mechanism, these secretion systems can be divided into two main categories: one-step and two-step delivery. In one-step delivery, including T4P, T1SS, T3SS, T4SS, T6SS, and T7SS, the substrates are directly transported from the bacterial cytosol to the extracellular space or the target cytoplasm. In two-step delivery, including T4P and T2SS, T5SS, T8SS, and T9SS, substrates are first transported to the periplasmic space between the IM and OM by the Sec/TAT pathway and then secreted into the extracellular space by a specialized OM secretion system. The Sec secretion system mainly transports unfolded secretory proteins to the outside of the cell membrane to enter a folded state without the consumption of energy [14]. The TAT secretion system mainly transports folded secretory proteins [15]. The two pathways are quite different in their mechanisms of action. T4P is involved in both one-step and two-step secretion [11]. All secretion systems (except T7SS) are present in Gram-negative bacteria, and several are used by Gram-positive bacteria, but they also have their special systems [16]. Mycobacteria are an exception because they have a membrane surrounding the cytoplasm and a unique bacterial membrane, thus requiring a one-step secretion system [17]. Abbreviation. ATP: adenosine triphosphate; ADP: adenosine diphosphate; C-U: chaperone–usher system; Sec: the general secretory; T4P: type IV pilus; TAT: twin-arginine translocation.

The bacterial secretion systems are important biological systems that play key roles. Through these systems, bacteria can release various compounds and proteins to interact with the external environment (Figure 1) [13]. In this section, we describe the structure, function, and mechanism of T5SS in detail.

### 2.1. T5SS

**Figure 2:**
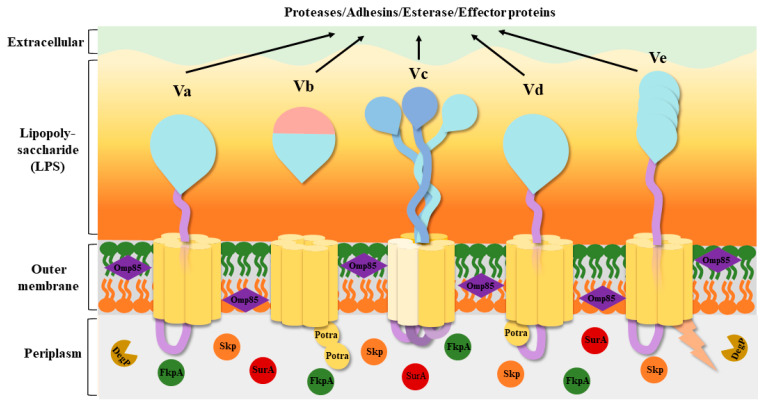
Structures of the five T5SS subtypes. The β-barrels are shown in yellow, the passengers are shown in blue, and the linkers connecting the β-barrel to the passenger are shown in light purple. The Omp85 family assembly factors are shown in dark purple. The periplasmic extension of Ve is shown in light orange. A number of periplasmic chaperones, SurA, Skp, FkpA, and DegP, are present in the periplasm, all of which promote the proper assembly of the β-barrel. The five subtypes have both similarities and differences in their structure. Vc requires three separate peptide chains to complete transport, forming three separate β-barrels through the connection between the peptide chains. Both Vb and Vd have POTRA structures; Vb has two, and Vd has only one. Their passengers are usually proteases, adhesins, esterase, and effector proteins. Abbreviation. Potra: polypeptide transport-associated; Va: classical autotransporters; Vb: two-partner passenger translocators; Vc: trimeric autotransporters; Vd: hybrid autotransporters; Ve: inverted autotransporters.

The secretion apparatus of T5SS is relatively simple, consisting of only one polypeptide chain. They are much smaller than most other secretion systems and span only one layer of the Gram-negative OM [18]. Because of the lack of energy in the periplasm and the absence of an ion gradient or proton gradient over the OM, T5SS seems to be able to transport toxins, adhesins, enzymes, and virulence factors without consuming energy [19]; hence, it is also known as the “autotransporter secretion system”. Currently, we know that the secretion of autotransporters requires the involvement of multiple factors [20], but the source of energy contributing to secretion remains unknown. T5SS has been roughly divided into five classes based on their domains and composition: classical autotransporters (Va), two-partner passenger translocators (Vb), trimeric autotransporters (Vc), hybrid autotransporters (Vd), and inverted autotransporters (Ve) (Figure 2) [21,22]. Some researchers have speculated that there is a sixth subtype (Vf) [23], but its structure and function are still unclear.

T5SS is composed of four identical domains: an N-terminal signal peptide sequence for translocation through the Sec pathway, a C-terminal β-barrel translocator domain [24], a linker domain, and a surface-fixed mature protein passenger domain that exerts biological activity in the extracellular space [25]. The β-barrel translocator domain was originally thought to extrude the passenger domain into the OM channel on the outside of the cell [26], promoting the secretion of soluble effectors across the OM [20], hence the name autotransporter. The surface-exposed passenger domains exhibit a diverse range of protease, adhesin, toxin, or receptor-binding activities that facilitate the formation of bacterial biofilms, enhance adhesion capabilities, and promote infection of host cells [27].

**Figure 3:**
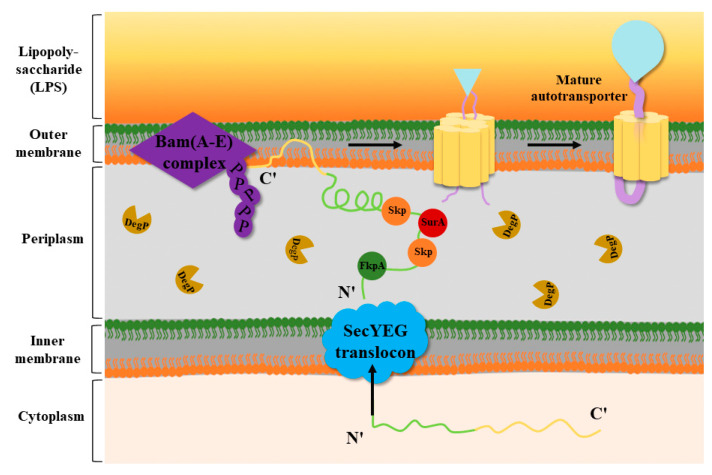
Transport mechanism of T5SS. In Gram-negative bacteria, the N-terminal signal peptide of the OM protein precursor is recognized by and binds to Sec molecules on the IM of the cell. The OM protein precursor enters the IM to arrive at the periplasm, and the N-terminal signal peptide is recognized and cleaved by signal peptidases. Subsequently, the OM protein that excises the signal peptide interacts with the periplasmic chaperones SurA, SkP, FkpA, and DegP. Among them, Skp, SurA, and FkpA can prevent β-barrel misfolding and assembly, and DegP can degrade misfolded proteins. With the help of Skp and DegP, the OM proteins are transported to the Bam complex and soon undergo OM trafficking to form a mature autotransporter [37].

Several factors, such as the periplasmic chaperones SurA, Skp, and DegP and the Bam complex (β-barrel assembly), are involved in the reaction with the OM proteins in the periplasm [28,29]. The working mechanism of T5SS is described in the following steps (Figure 3). Through the Sec pathway, the N-terminal signal peptide of the autotransporter proteins is inserted into the IM and transported to the periplasm [30], where it undergoes rapid OM trafficking after binding to the periplasmic chaperones SurA, Skp, and DegP. During transport, the β-barrel assembly of the Bam complex occurs [29]. SurA and Skp can prevent β-barrel misfolding and assembly, and SurA can promote correct β-barrel folding [31]. The BamA complex, a member of the Omp85 family, can facilitate the folding and insertion process of OM proteins [32]. It has five periplasmic POTRA domains that are critical for promoting BAM complex formation and interaction with substrate β-barrel proteins [33]. Knockout of the BamA homologs in *Escherichia coli (E. coli*) was found to cause misfolding of the β-barrel complex and bacterial cell death [34]. Secretory proteins are translocated through the β-barrel pore by interacting with at least one POTRA domain located on BamA or the translocase protein [18,35]. According to the physiological conditions of the protein itself and environmental factors, some proteins are still linked to the OM surface, while serine proteases such as Pet and EspP enhance the autocatalytic activity of the β-barrel, which cleaves the linker domain, releasing the passenger domain [25,36].

#### 2.1.1. Type Va Secretion: Classical Autotransporters

Subtype Va is the most typical T5SS. Many important virulence factors, such as the adhesin AIDA-I from *E. coli*, the protease IgA1 from Neisseria gonorrhoeae, and the lipase EstA from *Pseudomonas aeruginosa* (*P. aeruginosa*), are transported, processed, and secreted by type Va systems [38,39]. The Va system has four domains like the other isoforms, but it has a single structure. Meanwhile, in contrast to other subtypes, it has a long signal peptide sequence at the N-terminus that is recognized by the Sec proteins [40], allowing the secretory protein to be transported across the IM to the periplasm and promoting the correct folding of the protein [30]. In some cases, enzymatically active passengers can cleave off the passenger domain of type Va systems, thereby releasing the passengers into the extracellular medium while keeping the translocation domain anchored to the cell surface. However, noncleavable passengers typically possess adhesin functionality [18,41].

#### 2.1.2. Type Vb Secretion: Two-Partner Secretion Systems

The type Vb system is also known as the two-partner secretion (TPS) pathway. The structure of the Vb subtype is very different from that of the other subtypes; examples include the hyphal hemagglutinin FhaBC from *Bordetella pertussis*, ShlBA from *Serratia marcescens*, and the high-molecular-weight adhesins HMW1 and HMW2 from *Haemophilus influenza* (*H. influenza*) [42]. Its translocator domain and passenger domain are located on two different polypeptide chains [18]. The passenger domain is called TpsA, and the transport domain is called TpsB [43]. The genes encoding TpsA and TpsB proteins are organized into operons, and TpsA proteins possess TPS domains that are required for TpsB protein targeting and secretion [44]. The *TpsB* gene normally precedes the *TpsA* gene, and each TpsB protein has two POTRA domains that specifically recognize and translocate its cognate TpsA partner into the extracellular membrane space [45,46]. TPS plays an important role in the pathogenesis of bacteria by releasing virulence factors such as cytolysin and adhesin and promoting proteolysis, adhesion to host cells, biofilm formation, aggregation of bacteria, and survival. In particular, TPS can sense signals from changes in the host environment (temperature, iron, or inorganic phosphate) to regulate its expression [47].

#### 2.1.3. Type Vc Secretion: Trimeric Autotransporters

Subtype Vc systems are trimeric structures composed of three identical polypeptide chains, each providing four β-chains, thus forming three β-barrels by hydrogen bonding, which are then inserted into the OM with the help of the Bam complex. Each polypeptide chain offers a passenger domain, forming three extracellular passenger domains [48]. Unlike the single structure of subtype Va, they are usually described as trimeric autotransporter adhesins (TAAs), in which the head part of the passenger domain is involved in bacterial adhesion to host cells. They lack enzymatic functions and the ability of autoproteolytic cleavage for release from the cell surface. The most typical example is Yersinia adhesin A (YadA) from *Yersinia enterocolitica* and *Yersinia pseudotuberculosis* [49,50]. Other typical examples are the adhesin BadA from *Bartonella henselae* (*B. henselae*), SadA and SadB from *Salmonella typhimurium*, and Hia and Hsf from *H. influenzae* [18]. Although the monomeric structure of the Vc subgroup is similar to that of the Va subgroup, the C-terminal translocator domain of TAAs is the only conserved domain, while the N-terminal passenger domain consists of an extended stalk and an N-terminal head. Passenger domains are variable due to their different functionalities in different TAAs [25].

#### 2.1.4. Type Vd Secretion: Fused Two-Partner Secretion

Subtype Vd was not considered to belong to T5SS until the characterization of the patatin-like protein PlpD from *P. aeruginosa* in 2010. PlpD, whose folding mechanism and structure differ from those of other secreted passenger proteins, is the first active lipase found to be secreted through T5SS. Recombinant PlpD extracted from *E. coli* showed lipase activity, indicating its ability to recognize and catabolize lipids [51]. The phospholipase A1 autotransporter FplA from *Fusobacterium nucleatum* was found in 2017 [22]. Its 16 β-strands form a C-terminal β-barrel, which is similar to the β-barrel domain of the TpsB protein [18]. However, unlike the Vb type, Vd type autotransporters have only one POTRA domain. The passenger domain of Vd type autotransporters has only lipase activity and is cleaved upon completion of translocation [52,53].

#### 2.1.5. Type Ve Secretion: Inverted Autotransporters

Subtype Ve secretory proteins are known as “inverted autotransporters” because their structural order is inverted compared to that of subtype Va [54]; that is, the N-terminus of the protein is the translocator domain, and the C-terminus is the passenger domain that is transported to the extracellular space. Subtype Ve is found in the closely related adhesin intimin of *E. coli* and invasin of enteropathogenic *Yersinia* spp. [18]. Ve has a longer signal peptide than Va and prevents misfolding of the passenger domain. The translocator domain is the most conserved domain of the Ve type and is responsible for anchoring the protein into the OM and secreting the passenger domain. The passenger domain mainly secretes proteins into the extracellular space. In addition, it has a small periplasmic domain at the N-terminus of the peptide chain that is not found in other types [55]. In some conditions, the periplasmic domain assists in dimerization as well as in the interaction with peptidoglycan, forming a peptidoglycan binding motif that may anchor peptidoglycan and aid receptor interaction during host invasion [56].

### 2.2. Other Secretion Systems

In the above subsections, we have described the two-step T5SS in detail. In the following subsections, we introduce other secretion systems, which are classified according to the transport steps.

#### 2.2.1. One-Step Transport

Some secretion systems can transport proteins in one step from the cytosol to the extracellular space or target cells without a stopover in the periplasmic space. These include T1SS, T3SS, T4SS, T6SS, and T7SS. T1SS is widely found in Gram-negative bacteria. The T1SS apparatus is relatively simple, and its secretion is an extremely well-conserved one-step translocation process. T1SS is mainly composed of an ABC transporter protein in the IM, the periplasmic membrane fusion protein MFP, and a porin homologous to TolC-like receptors in the OM [57,58]. The most well-studied T1SS protein to date is hemolysin A from *E. coli* [59]. The function of T1SS is mainly to secrete toxins to enhance bacterial pathogenicity. There are eight T3SS subtypes, which are found in a variety of animal and plant pathogens [60]. T3SS is the most complex secretory system. T3SS is composed of apparatus proteins, chaperones, regulatory proteins, and effector proteins and is closely related to bacterial pathogenicity [61]. The structure of T3SS takes the shape of a “syringe”, mainly consisting of a basal body and a needle. The basal bodies, which are fixed to the IM and OM of the bacteria, are responsible for the recruitment and unfolding of proteins. The needle spans the bacterial and host cell membranes and can insert a pore in the host cell membrane through which the protein is subsequently transported and reaches the host cell [62]. According to their functions and genomic coding, T4SS can be divided into three subtypes, types IVa, IVb, and IVc, which are widely distributed in bacteria [63]. Type IVa can be divided into four subunits according to their functions: a type 4 coupling protein (T4CP), an IM complex (IMC), an OM complex (OMC), and cell surface pili or adhesins [64]. In contrast to other secretion systems, T4SS can exchange substances between cells [65]. Through bacterial conjugation transfer, DNA release and uptake, and secretion of effector proteins, bacteria can improve their resistance to the external environment. T4SS can transfer DNA from one bacterial cell to another. In this process, T4SS first binds plasmid DNA to a transporter protein complex and then delivers the DNA-protein complex from the donor cell to the recipient cell. This is the main pathway for horizontal gene transfer in bacteria. Some bacteria can also release DNA directly into the environment via T4SS or take DNA from the environment. Methods of DNA release and uptake can increase the genetic diversity of bacteria, helping them adapt to changing environments. In addition to DNA, T4SS can secrete proteins. These proteins can be involved in DNA transfer or become bacterial virulence factors secreted into the host cell, interfering with its normal function [63,64,65,66]. T6SS is widely found in Gram-negative bacteria. The T6SS complex is composed of a variety of proteins. The apparatus can be divided into two substructures: a membrane complex (MC) and a baseplate complex (BC) [67,68]. In contrast to other secretion systems, T6SS can secrete large amounts of various toxins into target cells or the environment to attack, and it can secrete protective proteins to protect itself from accidental injury, thereby also competing for nutrients [69]. T7SS, first discovered in *Mycobacterium tuberculosis*, only exists in Gram-positive bacteria [70]. T7SS is a new secretion pathway that can replace the Sec or TAT pathway. In Gram-positive bacteria, some proteins without typical signal peptide sequences cannot be transported to the periplasm via the Sec or TAT pathways, so T7SS replaces them as a novel pathway for secreting proteins. T7SS is composed of two parts: cytosolic components (EspG, EccA) and membrane proteins (EccB, EccC, EccD, EccE, and MycP) [71]. The EspG protein is conserved, and the main T7SS chaperone is only present in the cytoplasm and is essential for substrate secretion. EccA belongs to the ATPase family associated with diverse cellular activities. EccB may be involved in the assembly and stabilization of T7SS, and EccC is an ATPase that provides energy for protein transport or recognition of secreted substrates. EccD, a transmembrane protein with 11 transmembrane domains, may be involved in the formation of translocation channels. EccE is a glycosyl transferase protein with two transmembrane domains and a C-terminal cytosolic domain. EccD can form critical interactions with EccE or EccC to enhance structural stability. MycP is known to be a subtilisin-like serine protease that cleaves substrate proteins such as EspA, EspB, and EspC to aid their secretion and is closely related to protein and T7SS stabilization [72]. T7SS is primarily involved in the assembly, the secretion of protein polymers, and immune escape and is essential for bacterial virulence [73].

#### 2.2.2. Two-Step Transport

Some proteins are transported in two steps, first across the IM with the help of the Sec or TAT pathway into the periplasmic space, where they remain for a period of time. In the periplasmic space, proteins undergo correct folding and modification, which is critical for protein stability and function. Some misfolded or modified proteins can be recognized and degraded by periplasmic chaperones. Erroneous proteins may not pass through the OM or may not function outside the cell. At the same time, in the periplasm, proteins can be directed to the correct position and then across the OM by the secretion systems. The secretion systems that play a role here include T2SS, T5SS, T8SS, and T9SS. The secretion apparatus of T2SS is a complex structure composed of 12–15 secretory proteins [74]. Its substrates include various toxins and hydrolases, and its main functions are to absorb nutrients and release toxins [75]. T2SS helps bacteria adapt to different external environments by facilitating the specific transport of folded proteins to the extracellular space via special OM channels and binding to host cells. T8SS, also known as the nucleation/precipitation pathway, mainly secretes extracellular amyloid fibers [76]. It can act as an adhesin and as a structural framework to promote biofilm formation and escape the killing effect of complement factors in the host immune system [77]. T9SS was recently discovered in *Flavobacterium johnsoniae* and *Porphyromonas gingivalis* [78]. It can secrete virulence factors, various hydrolytic enzymes, and adhesins through the OM, mediate gliding motility, and evade the host immune system. It performs protein transfer to the IM with the help of the Sec system [79,80].

## 3. The Love and Hate Relationship

Intelligent diderm bacteria secrete many effector proteins to compete for survival, resist the host immune system, launch attacks on host cells, and perform a series of activities such as adhesion, invasion, colonization, and dissemination. These effector proteins are transported across the membrane with the help of secretion systems that are not separate but have more or less “emotional” relationships with each other, either “love” or “hate”. Here, the “love and hate relationship” represents the interaction of T5SS with other secretion systems. “Love” represents their cooperation or complementary relationship, and “hate” represents their inhibition or antagonism. In this section, we discuss the “love and hate relationship” between T5SS and other secretion systems. To date, T5SS has been found in Gram-negative bacteria, and T7SS has only been found in a few Gram-positive bacteria [70]. They are still two parallel lines that have no connections for the time being, so no “emotional” analysis will be performed on them. Meanwhile, there is also no evidence that T5SS interacts with T8SS and T9SS.

### 3.1. Between T5SS and T1SS

Unlike T3SS, T4SS and T6SS transport effector proteins to target cells in one step with no stopover in the periplasm. T1SS transports the substrate only to the external environment [9]. T1SS is a highly specific secretion system that secretes specific proteins, such as toxins and enzymes, for infection and interaction [81]. Although the exact relationship between T1SS and T5SS is not clear, some studies have found that stimulation with a high concentration of the signaling molecule cyclic diguanylate (c-di-GMP), changing the status of T1SS and T5SS, induces the secretion of adhesins and upregulates T6SS, which affects the formation of bacterial biofilms, driving a bacterial lifestyle switching from motile to sessile. The second messenger, c-di-GMP, is widely present in different stages of bacterial development. In bacterial pathogenic mechanisms, the expression of secretion system components and/or effector proteins is regulated by c-di-GMP. As the concentration of c-di-GMP increases, the adhesin LapA is synthesized and secreted in T1SS. LapA is translocated from its C-terminal to the OM pore LapE by the membrane fusion protein LapC and the ABC transporter LapB, leading to the formation of a biofilm. Upon biofilm formation, the regulatory protein LapD binds to c-di-GMP to induce sequestration of the periplasmic protease LapG, preventing LapA from being cleaved. In T5SS, CdrA and CdrB are also regulated by c-di-GMP levels. CdrA and LapA are functionally similar but not homologous, whereas LapD-G is. T6SS has been associated with bacterial biofilm expression. The secreted protein Hcp1 is a marker of T6SS activity. When the level of c-di-GMP increases, the expression of Hcp1, the activity of T6SS, and the biofilm formation capacity increase. Although T1SS, T5SS, and T6SS are distinct systems, they share a common regulatory molecule in c-di-GMP, suggesting a coordinated response to environmental cues. This coordination may be crucial in the context of bacterial pathogenesis, where the ability to adhere to host cells and form biofilms can significantly affect the course of infection [82].

### 3.2. Between T5SS and T2SS

T2SS is widely found in Gram-negative bacteria. Like T5SS, it is a two-step secretion system; they both depend on the Sec/TAT pathway to secrete substrates. Many studies have identified T2SS as a pivotal factor in bacterial pathogenicity in both animals and plants. T2SS spans across the bacterial envelope and utilizes pseudopilus structures to facilitate the export of substrates through OM secretion channels [83]. It has been suggested that surface localization of lipoproteins is rare in diderm bacteria. For example, in *Gammaproteobacteria*, partially hydrophobic acylated proteins overcome the obstacles in the surrounding space with the help of the lipoprotein-specific chaperone pathway, and T2SS and T5SS are involved in the secretion of *Gammaproteobacteria*-specific surface lipoproteins [84]. Other studies have shown that T2SS and T5SS cooperate with T1SS, T4SS, and T9SS, acting as “vanguards” to secrete large adhesin proteins that attach to the bacterial cell surface and extend outward to mediate adhesion to host cells, while the remaining systems provide “reserve forces,” providing subtle but highly flexible adhesins such as fimbriae and flagella to enable further enhancement of bacterial adhesion [11].

### 3.3. Between T5SS and T3SS

The composition of T3SS is very complex; it is like a “syringe” and can directly inject effector proteins from bacterial cells into host cells. Its immune escape ability plays an important role in the invasion of host cells [60]. It has been reported that during infection with some intestinal pathogens, some T5SS cooperate with T3SS to establish contact with cognate adhesin receptors found on the surface of pathogens and hosts, thereby causing reorganization of the host cytoskeleton or invasion into cells and enhancing its intracellular motility to evade phagocytosis or form new structures to protect bacteria [85].

During EPEC infection, T5SS secretes an EspC autotransporter that is then efficiently transferred into epithelial cells by a T3SS translocon, translocated intimin receptor (Tir), causing cytotoxic damage [86]. Tir is a key EPEC and EHEC effector protein and causes A/E lesions by interacting with the bacterial type Ve protein intimin. A/E lesions are characterized by tight attachment of bacteria to host cells, resulting in the disappearance of host cell microvilli and the formation of an actin “pedestal”. This pedestal structure is due to a strong rearrangement of the host cytoskeleton, particularly the aggregation of actin at the site of bacterial attachment. Meanwhile, Tir can activate host cell signaling pathways. This alteration contributes to bacterial adhesion and viability and is also responsible for diarrhea [87,88].

Other studies have discovered that the adhesin activity of IcsA secreted by the T5SS of *Shigella* was altered when stimulated by T3SS [89,90]. The interaction between T3SS and T5SS was demonstrated by disruption of a single cysteine residue near the N-terminus of IcsA, the passenger, which resulted in a loss of adhesion ability that was subsequently restored by expression in the background of a constitutively active T3SS [91,92].

### 3.4. Between T5SS and T4SS

T4SS is also a complex protein secretion system capable of transporting DNA. In *B. henselae*, the adhesin BadA is secreted into the external environment by T5SS and translocated by T3SS and T4SS [93,94]. Another virulence factor is the T4SS core VirB/D-like subcomplex. This structure requires the VirB1-11 protein assembly to form the secretion machinery and T4P, while the VirD4 protein is responsible for lifting the substrate to T4SS for secretion through the translocation channel. But when BadA is active, the functions of VirB/D-like complexes are suppressed and vice versa. This is because the presence of BadA creates a physical distance between the OM of the pathogen and the host cell membrane through its effective length. The distance hinders the transport of VirB/D4 and thus reduces pathogen virulence. Because of this antagonism, VirB/D4 has almost zero retention value in the presence of BadA, and most human *B. henselae* isolates are lost to either BadA or VirB/D4. Overall, the interaction of the BadA and VirB/D-like complexes in *B. henselae* reveals a possible antagonistic relationship between T4SS and T5SS that may influence pathogen virulence and infectivity [95,96].

### 3.5. Between T5SS and T6SS

T6SS is widely found in Gram-negative bacteria. Its structure is similar to that of bacteriophages, including a base, a tail tube, and a tip complex. It passes through the bacterial membrane before injecting secreted substances into the host cell [97]. *Pseudomonas aeruginosa* has very powerful molecular weapons for killing or weakening other microorganisms. It has two main methods: contact-dependent killing and contact-independent killing. Contact-dependent killing can act by direct delivery to an effector of a competitor. This process is usually mediated by the specialized secretion systems T5SS and T6SS. Proteins secreted by T5SS can mediate contact-dependent killing through direct interactions with neighboring bacteria. The CdiA protein secreted by T5SS harbors nuclease activity and inhibits bacterial cell growth by degrading the tRNA or DNA of the host cell. *Pseudomonas aeruginosa* uses its T6SS to deliver toxic effectors to neighboring bacteria, causing their lysis and death. The effector proteins of T6SS, such as Hcp, VgrG, and PAAR, have different functions. Some have amidase or glycoside hydrolase activity and are capable of degrading bacterial peptidoglycan scaffolds. Some are phospholipases that alter the composition of lipid bilayers and cause cell lysis. Contact-independent killing typically involves other secretion systems that secret soluble factors such as bacteriocins and other small antimicrobial molecules that can diffuse through the environment and affect distant cells [98,99,100]. Rhs from *Dickeya dadantii* is an effector secreted by T6SS, which has a conserved central region and variable N- and C-terminal regions [101]. The 3′ region of rhs encodes a recombination of a C-terminal toxin tRNase domain and its downstream gene, while the 5′ end of rhs encodes a T5SS contact-dependent inhibition (CDI) passenger domain, which enables cell surface expression and transfer of the C-terminal toxic effector domain to inhibit the growth of *E. coli* prey cells [102,103]. In Gram-negative bacteria, both T5SS and T6SS secrete toxins to the extracellular space, which antagonize other microorganisms and thus seize the living environment [104].

### 3.6. Between Secretion Systems and Virulence Factors

The secretion systems play an important role in the secretion, activity, and stability of virulence factors. T1SS usually secretes bacteriocins and adhesins to spread into host cells. Meanwhile, it also secretes virulence proteins such as iron clearance proteins, lipases, proteases, and perforins, which are conducive to nutrient capture, greatly improving the invasiveness of bacteria [105]. T2SS mainly secretes proteases, lipases, adhesins, and exotoxins to compete for nutrients, destroy host cells, and form biofilms. After T2SS releases plant cell-wall-degrading enzymes to destroy the cell wall of plant cells, T3SS directly injects effector proteins into host cells based on its characteristics [106]. However, T3SS mainly secretes adenylate cyclases and phospholipases, directly transporting virulence factors into cells, increasing cytotoxicity, inducing apoptosis, and promoting bacterial colonization and invasion [107]. T4SS can transport individual proteins as well as protein–protein and DNA–protein complexes, it is a tool for DNA transfer, and it can enhance bacterial drug resistance [108]. T5SS secretes autotransporters to cause cytotoxicity through translocating intimin receptor Tir, which is mediated by T3SS, and its substrates are mostly proteases, lipases, and adhesins, which can enhance bacterial pathogenicity [85]. T6SS has shown outstanding performance in biofilm formation, DNA transport, extracellular metal uptake, and antifungal activity. The virulence factors secreted by T3SS and T6SS are also involved in host immune regulation [109,110]. T7SS not only secretes virulence proteins, maintains Zn^2+^ homeostasis, forms biofilms, and maintains motility but also promotes adhesion to the host and escapes immune responses, which plays a crucial role in promoting long-term bacterial survival [111]. The T7SS substrate EsxA is required to delay the apoptosis of epithelial cells and dendritic cells infected with *Staphylococcus aureus* [112]. In addition, T8SS secretes some adhesins to promote biofilm formation [77]. T9SS secretes motor proteins, adhesins, proteases, and chondroitinases to improve gliding motility; however, gliding motility is also dependent on motile adhesins [113].

## 4. Conclusions

So far, eleven secretion systems (T1SS–T11SS) have been reported. However, T10SS and T11SS have been poorly studied. Thus, nine secretion systems (T1SS–T9SS) are covered in this article. Secretion systems can be divided into two categories based on their secretion steps: one-step and two-step delivery. T4P, T1SS, T3SS, T4SS, T6SS, and T7SS belong to one-step delivery, where the substrate from the bacterial cytoplasm is transferred directly to the target cell. In two-step delivery, including T4P and T2SS, T5SS, T8SS, and T9SS, the substrate is first transported to the periplasmic space between the IM and OM by the Sec/TAT pathway and then secreted to the OM by the secretion system. Among various secretion systems, there is a particular secretion system, T5SS, which is well known as the “autotransporter”. Its structure is very simple and is composed of four identical domains: an N-terminal signal peptide sequence, a C-terminal β-barrel translocator domain, a linker domain, and a passenger domain. T5SS has five subtypes, subtypes Va–e, which share similarities and differences in structure, and their secreted effectors are usually proteases, adhesins, esterases, and effector proteins. Then, the interplay between T5SS and other secretion systems is presented. T5SS and T1SS play a role in promoting biofilm formation under the regulation of c-di-GMP levels. Together, T5SS and T2SS are involved in the secretion of specific proteins in *Gammaproteobacteria*. Moreover, they also cooperate with T1SS, T4SS, and T9SS to secrete large adhesion proteins that promote bacterial adhesion. Some T5SS cooperate with T3SS not only to enhance bacterial adhesion but also to cause reorganization of the host cytoskeleton or invasion of cells, to enhance their intracellular motility to escape phagocytosis, or to form new structures to protect the bacteria. BadA, which is secreted by T5SS, reduces T4SS activity and reduces bacterial virulence. Both T5SS and T6SS can compete with other microorganisms by performing contact-dependent killing. In addition, there are many connections between secretion systems and virulence factors. All of them have a significant impact on the pathogenicity of bacteria.

## 5. Future Perspectives

Bacterial secretion systems are an indispensable part of bacterial life activities, and their importance is self-evident. Under certain conditions, secretion systems act synergistically, while under other conditions, antagonism between secretion systems occurs. Moreover, the secretion systems exert a significant impact on immune regulation to ensure that bacteria can invade the host and multiply. Many bacteria deliver toxins or effector proteins directly into host cells via secretion systems, and these effectors can interfere with host cellular signaling and suppress immune responses. Some bacteria can evade the clearance of the immune system by inducing immune tolerance through the effectors secreted by secretion systems, and some effectors can also trigger the inflammatory response of the host. In addition, the secretion system can deliver effector proteins into host cells, causing apoptosis [114,115]. For example, T3SS manipulates *P. aeruginosa* to release inflammatory responses and evade phagocytosis by phagocytes to survive in the host [116]. Hemolysin coregulated protein (Hcp) secreted by T6SS in *Helicobacter pullorum* can cause apoptosis of host cells [117].

Unfortunately, few studies have been conducted on secretion systems, and even fewer have been conducted to analyze the interactions between them. New secretion systems and pathways have been discovered, which shows that bacteria are more complex than people often imagine. Moreover, it also implies that we need to conduct in-depth research on secretion systems to reveal the mysteries of biological characteristics, pathogenic mechanisms, and drug resistance of bacteria as soon as possible. We expect that when our understanding of bacteria is more mature, the medical technology to combat bacterial diseases will be further improved.

## Data Availability

All datasets generated for this study are included in the article.
